# Supplementary material for: VIRIDIC—A Novel Tool to Calculate the Intergenomic Similarities of Prokaryote-Infecting Viruses
Source: Viruses. 2020 Nov 6;12(11):1268. doi: 10.3390/v12111268 (PMC7694805; doi:10.3390/v12111268)
Supplement: Supplementary file 1 [file viruses-12-01268-s001.pdf]

viruses

MDPI

Supplementary Materials: VIRIDIC—A Novel Tool to Calculate the Intergenomic Similarities of Prokaryote-Infecting Viruses

Table 1. The complete list of viral genomes used for benchmarking VIRIDIC, their genome properties and intergenomic similarity values produced by different tools.

Phage.name	RefSeq.No.	Size..bp .	mol.G. C	NCBI BLAST N	Emboss Stretcher	PASC	Gegenees BLASTN_ 5	Gegenees BLASTN_0	Ortho_ANI	SDT	VIRIDIC
Citrobacter phage CR44b	NC_023576.1	39,207	50.5	24.22	53.9	45.01	23.38	0	64.41	56.3	26.39
Citrobacter phage CR8	NC_023548.1	39,651	49.7	24.17	55.1	45.45	30.89	0	64.7	56.2	26.63
Citrobacter phage phiCFP-1	NC_028880.1	38,625	50.3	59.98	51.5	62.49	47.64	0	73.34	69.4	59.791
Citrobacter phage SH1	NC_031066.1	39,434	51	51.59	63.6	60.15	39.42	0	72.87	67.4	57.173
Citrobacter phage SH2	NC_031092.1	39,158	50.7	56.77	63.2	59.76	40.18	0	72.06	66.7	55.73
Citrobacter phage SH3	NC_031123.1	39,444	50.6	24.96	55.7	44.98	33.51	0	64.32	56.9	25.893
Citrobacter phage SH4	NC_031018.1	39,274	52.6	23.43	55.3	43.9	32.43	0	64.38	56.6	26.457
Cronobacter phage Dev2	NC_023558.1	38,966	52.6	22.91	55.1	44.89	33.26	0	65.37	56.7	26.621
Delftia phage IME-DE1	NC_028702.1	38,084	60.4	8.49	47.5	31.77	0	0	61.81	51.3	11.394
Enterobacter phage E-2	NC_029102.1	36,051	50.6	49.61	47.6	60.76	41.59	0	74.81	41.3	57.395
Enterobacter phage E-3	NC_028795.1	31,522	50.8	44.79	47.6	56.46	44.88	0	72.98	41.5	54.622
Enterobacter phage phiEap-1	NC_028772.1	39,133	51.7	45.02	58.1	53.92	35.89	0	69.66	64.2	49.298
Erwinia phage FE44	NC_022744.1	39,860	48.6	51.64	61.5	58.45	31.2	0	69.84	64.9	54.021
Erwinia phage vB_EamP-L1	NC_019510.1	39,282	51.9	42.79	56.7	50.36	40.86	0	67.64	62.4	43.245
Escherichia phage 13a	NC_011045.1	38,841	48.4	85.95	85.8	86.43	83.18	74.17	95.12	91.8	86.709
Escherichia phage 285P	NC_015249.1	39,270	48.7	50.66	60.2	54.45	34.75	0	69.78	64.8	53.489
Escherichia phage 64795_ec1	NC_031114.1	39,235	48.7	83.83	84.5	85.81	78.6	64.77	92.4	89.3	84.971
Escherichia phage BA14	NC_011040.1	39,816	48.8	49.39	62	54.98	39.83	0	69.57	64.7	51.881
Escherichia phage CICC 80001	NC_027387.1	38,810	48.8	83.42	66.3	84.38	76.75	64.68	92.23	91.3	83.853
Escherichia phage EcoDS1	NC_011042.1	39,252	49.9	24.09	54.3	43.42	16.27	0	66.4	56.7	27.415
Escherichia phage K1F	NC_007456.1	39,704	49.8	25.54	54.6	43.39	44.12	0	66.32	56.8	25.98
Escherichia phage K30	NC_015719.1	40,940	51.4	45.51	58.2	52.83	34.56	0	70.39	64.9	49.317
Escherichia phage LM33_P1	NC_031937.1	38,979	50.2	24.14	54.8	44.49	28.05	0	64.63	56.1	26.636
Escherichia phage P483	NC_028822.1	40,829	48.4	48.68	47.9	55.98	34.93	0	69.87	40.9	51.325
Escherichia phage P694	NC_028863.1	40,477	48.4	51.07	59	57.81	39.22	0	68.68	64.5	54.033

Escherichia phage PE3-1	NC_024379.1	39,093	49.9	23.57	54.3	44.88	28.99	0	64.65	57.1	26.39
Escherichia phage T3	NC_003298.1	38,208	49.9	68.32	71.3	68.02	72.32	27.26	81.35	75.6	66.165
Escherichia phage T7	NC_001604.1	39,937	48.4	100	100	100	100	100	100	100	100
Escherichia phage vB_EcoP_GA2A	NC_031943.1	40,470	51.1	22.99	55.6	43.88	21.63	0	64.24	55.6	25.346
Klebsiella phage K11	NC_011043.1	41,181	53.2	43.84	58.1	51.89	30.33	0	70.58	64.4	47.067
Klebsiella phage K5	NC_028800.1	41,698	52.5	44.21	57.6	53.31	29.91	0	70.48	64.6	48.382
Klebsiella phage KP32	NC_013647.1	41,119	52.4	45.02	58.9	52.89	35.94	0	70.44	64.7	49.747
Klebsiella phage vB_Kp1	NC_028688.1	40,114	53.3	44.79	51.5	54.85	32.73	0	71.02	64	49.106
Klebsiella phage vB_KpnP_KpV289	NC_028977.1	41,054	52.6	44.82	58.7	53.32	32.06	0	71.16	64.1	49.076
Kluyvera phage Kvp1	NC_011534.1	39,472	48.6	49.54	60.7	55.5	36.05	0	70.23	64.8	52.401
Morganella phage MmP1	NC_011085.3	38,457	46.5	41.21	58.1	49.15	38.39	0	67.28	60.1	41.664
Morganella phage vB_MmoP_MP2	NC_031115.1	39,616	46.9	36.16	58.3	47.96	28.14	0	69.1	59.2	37.78
Pelagibacter phage HTVC019P	NC_020483.1	42,084	34	0	47.1	18.13	0	0	0	42.9	0.336
Pseudomonas phage gh-1	NC_004665.1	37,359	57.4	19.09	52.2	40.75	0	0	64.53	55	21.748
Pseudomonas phage Pf-10	NC_027292.1	39,167	56.5	19.46	53.4	39.7	21.96	0	66.04	53.7	21.826
Pseudomonas phage phi15	NC_015208.1	39,562	58.2	18.7	52.6	37.56	16.27	0	63.8	52.8	19.349
Pseudomonas phage phiIBB-PF7A	NC_015264.1	40,973	56.3	21.43	51.7	37.65	0	0	63.78	54.1	21.646
Pseudomonas phage phiPSA2	NC_024362.1	40,472	57.4	19.88	53.2	39.74	0	0	64.12	54.4	20.97
Pseudomonas phage Phi-S1	NC_021062.1	40,192	56.2	21.27	52.1	39.42	0	0	65.1	54.1	22.328
Pseudomonas phage PPPL-1	NC_028661.1	41,149	57	18.92	53.3	39.3	0	0	65.4	53.7	20.509
Pseudomonas phage PPpW-4	NC_023005.1	41,386	56.8	18.15	52.5	38.18	0	0	63.7	52.8	19.323
Ralstonia phage RSB2	NC_023736.1	40,411	61.8	6.84	49.5	31.18	0	0	62.42	49.2	9.229
Salmonella phage BP12A	NC_031258.1	39,696	49	49.68	61.1	56.65	38.39	0	68.08	64.3	52.903
Salmonella phage phiSG-JL2	NC_010807.1	38,815	50.9	51.58	63.1	58.42	42.83	0	72.51	67.5	57.31
Salmonella phage Vi06	NC_015271.1	38,368	48.9	69.09	71.4	70.8	66.73	26.13	85.86	81.1	71.469
Stenotrophomonas phage IME15	NC_019416.1	38,513	53.7	63.03	68.5	66.6	45.93	6.92	78.24	72.8	65.795
Vibrio phage ICP3	NC_015159.1	39,162	42.9	22.95	52.5	38.02	30.99	0	63.74	53.4	25.783
Vibrio phage N4	NC_013651.1	38,497	42.8	23.52	51.4	38.09	28.65	0	63.12	53.7	25.397
Vibrio phage VP4	NC_007149.1	39,503	42.6	22.74	51.9	37.94	25.27	0	63.99	54.1	24.695
Yersinia pestis phage phiA1122	NC_004777.1	37,555	48.3	85.46	86.1	87.57	82.87	75.35	93.72	91.7	87.804
Yersinia phage Berlin	NC_008694.1	38,564	47.2	49.52	59.1	55.32	32.7	0	69.67	64.3	52.311
Yersinia phage phiYeO3-12	NC_001271.1	39,600	50.6	50.22	63	58.14	42.87	0	72.78	67	55.5
Yersinia phage vB_YenP_AP10	NC_028655.1	39,235	51.8	52.04	63.1	59.77	42.73	0	71.04	66.7	55.146
Yersinia phage vB_YenP_AP5	NC_025451.1	38,646	50.7	50.08	62.8	60.11	39.27	0	73.98	67.4	55.957
Yersinia phage Yepe2	NC_011038.1	38,677	47.3	49.53	61.2	55.18	33.41	0	69.77	64.2	52.021
Yersinia phage Yep-phi	NC_023715.1	38,616	47.1	49.15	52.6	56.72	34.64	0	70	64.2	52.106
